# High throughput screening of CO_2_-tolerating microalgae using GasPak bags

**DOI:** 10.1186/2046-9063-9-23

**Published:** 2013-12-17

**Authors:** Zheng Liu, Fan Zhang, Feng Chen

**Affiliations:** 1 Institute of Marine and Environmental Technology, University of Maryland Center for Environmental Science, 701 E Pratt St, Baltimore, MD 21202, USA

**Keywords:** CO_2_ sequestration, Microalgae, High through-put selection

## Abstract

**Background:**

Microalgae are diverse in terms of their speciation and function. More than 35,000 algal strains have been described, and thousands of algal cultures are maintained in different culture collection centers. The ability of CO_2_ uptake by microalgae varies dramatically among algal species. It becomes challenging to select suitable algal candidates that can proliferate under high CO_2_ concentration from a large collection of algal cultures.

**Results:**

Here, we described a high throughput screening method to rapidly identify high CO_2_ affinity microalgae. The system integrates a CO_2_ mixer, GasPak bags and microplates. Microalgae on the microplates will be cultivated in GasPak bags charged with different CO_2_ concentrations. Using this method, we identified 17 algal strains whose growth rates were not influenced when the concentration of CO_2_ was increased from 2 to 20% (v/v). Most CO_2_ tolerant strains identified in this study were closely related to the species *Scenedesmus* and *Chlorococcum*. One of *Scenedesmus* strains (E7A) has been successfully tested in in the scale up photo bioreactors (500 L) bubbled with flue gas which contains 10-12% CO_2_.

**Conclusion:**

Our high throughput CO_2_ testing system provides a rapid and reliable way for identifying microalgal candidate strains that can grow under high CO_2_ condition from a large pool of culture collection species. This high throughput system can also be modified for selecting algal strains that can tolerate other gases, such as NOx, SOx, or flue gas.

## Background

Increasing atmospheric greenhouse gas emission by human activities has been regarded as a major challenge of global sustainability. CO_2_ is a primary greenhouse gas, which makes up approximately 83.6% of the total greenhouse gas emission [1].

Increasing level of CO_2_ causes global warming and the subsequent environmental issues such as the rising sea level and snow or ice melting [2,3].

Biological fixation carried out by photosynthetic plants and microalgae has attracted increasing attention as an environmentally friendly CO_2_ mitigation strategy [4,5]. Photosynthesis renews oxygen in the atmosphere while fixing CO_2_ into potentially useful biomass. Microalgae are emerging as a promising biological fixation system; each acre of microalgae is able to fix three to five times more CO_2_ than the same area of terrestrial plants [6]. Meanwhile, microalgae are also able to remove nitrogen, phosphorus, and heavy metals from wastewater, and algal biomass can be converted into useful products, such as biofuels, nutraceutical products, animal feed and fodder for aquaculture [7-9].

Exhaust gases from power plants attribute to ca. 40% of the U.S. annual CO_2_ emission in 2010 [10]. Earlier studies have reported that microalgae can be used to sequester CO_2_ in power plant flue gases [11-13]. The concentration of CO_2_ in power plant exhausts varies from 10-15% depending on the source of fuels [10]. Therefore, the ideal microalgal candidates for sequestering CO_2_ in flue gases should be able to grow under CO_2_ concentration above 10%. It is known that different species of microalgae can tolerate different levels of CO_2_. For examples, it has been reported that *Chlorella sp.* and *Euglena gracilis* can tolerate up to 40% CO_2_[14], *Chlorococcum littorale* could endure 60% CO_2_[15], *Scenedesmus sp.* could grow under 80% CO_2_[14], and *Cyandium caldarium* were successfully grown under 100% CO_2_[16]. However, it is difficult to evaluate and compare the actual growth rates of these algae under high levels of CO_2_ because some strains just tolerate but do not grow under high CO_2_ condition. Moreover, a comparison within the same species in different studies can also be challenging due to the different experimental setup. It has been reported that *Scenedesmus obliquus* can tolerate up to 18% CO_2_, but the optimal growth was observed with 6% CO_2_[17]. It is also reported that *S. obliquus* grew successfully under 70% CO_2_, however, the highest growth rate occurred below 10% CO_2_[18]. Nevertheless, in a separate study, the optimal growth of *S. obliquus* at 15% CO_2_ was observed [19]. The inconsistency of these results may be caused by the difference in the experimental setup. Many earlier studies conducted the CO_2_ tolerance tests using flasks bubbled with certain levels of CO_2_. In this case, the actual concentration of CO_2_ that algae are exposed to is hard to monitor because a certain amount of CO_2_ can be lost to air due to bubbling [20]. Difference in light, temperature, culture media and containments, bubbling rates, and other factors may all contribute to variable CO_2_ tolerance within the same species [21,22].

Microalgae are diverse in the natural environment. It has been estimated that about 200,000-800,000 algal species exist in nature, of which about 35,000 species have been described [23,24]. Thousands of algal strains have been isolated, characterized and maintained in different laboratories and culture collection centers. To select the candidates that have a high CO_2_ affinity from this large pool of algal collections can be very time-consuming and technical challenging, particularly when the growth of algae need to be measured. A high throughput method for evaluating the capability of these algal strains for CO_2_ tolerance could facilitate the research in algal sequestration of CO_2_ pollutions.

In this study, we designed a high throughput system to select microalgae based on their CO_2_ tolerating capability. The system we described here includes a CO_2_-air mixture device that provide a desired CO_2_ level, and a DB GasPak™ EZ bags to hold the CO_2_ gas. Microalgal cultures can be dispensed into a 24, 48, or 96-well plate that will be incubated inside the GasPak™ bag. This high throughput system can also be charged with flue gas. It provides a high-throughput, uniform and repeatable method for CO_2_ tolerant strains selection and comparison. Using this system, we identified 17 strains of microalgae from our culture collection that can grow under 20% CO_2_ condition.

## Results and discussion

**Figure 1:**
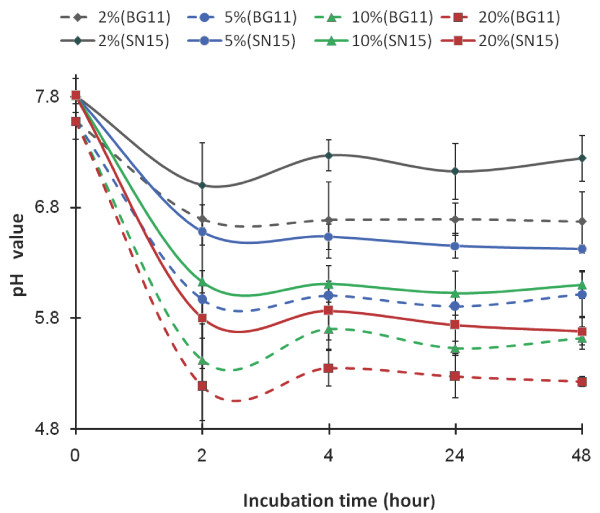
**Time course of pH values of BG11 (…) and SN15 (—) medium after incubation in 2, 5, 10 and 20% CO**_**2**_**.** The pH values were measured after the plates were exposed to different CO_2_ concentration for 2, 4, 24 and 48 hours.

The pH value of the medium is used as a direct indicator of the ambient CO_2_. To test the stability of GasPak bags for holding desired CO_2_ concentrations during the incubation time, we monitored pH in the wells of microplates over a two-day period. We monitored pH for 2 days because the GasPak bags will be opened for OD reading every 48 hours and recharged with CO_2_. The pH values in the medium dropped sharply and reached equilibriums within 2 hours after the bags were filled with CO_2_ (Figure 1). The value of pH reached different equilibriums depending on the percentage of CO_2_ charged to the bags, suggesting that the system is sensitive to a small difference in CO_2_ input. In all the treatments, CO_2_ levels remained relatively constant in 48 hrs, suggesting that the GasPak bag can provide a stable environment to test the effect of different concentrations of CO_2_ on the algal growth. When charged with same amount of CO_2_, the marine medium (SN15) was able to maintain higher pH values compared to the freshwater medium (BG11). For example, under 20% CO_2_, pH in the BG11 medium is 5.2, while pH in the SN15 medium is 5.7. It has been known that seawater is a good buffering system, and the solubility of CO_2_ decreases when the salinity increases [25].

**Figure 2:**
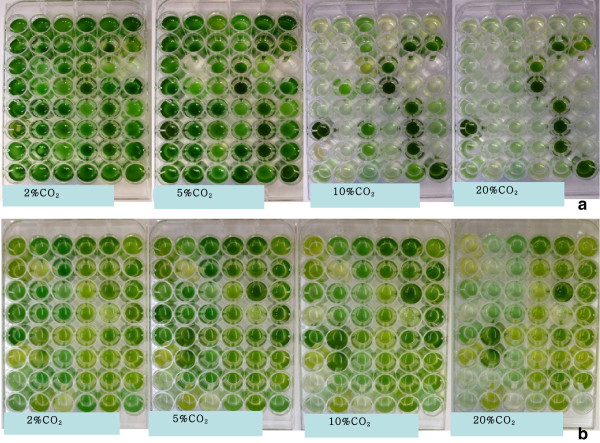
**Top view of algal cultures in microplates after exposing to different concentrations of CO**_**2 **_**(2, 5, 10 and 20% v/v respectively), (a) strains grown in BG11 medium and (b) strains grown in SN15 medium.**

The GasPak bag system provides a uniform environment for testing many algal strains at the same time. After 14 days, distinct growth performance of different algal strains can be visualized (Figure 2). At the lower CO_2_ level (2%), nearly all the algal strains grew well and showed healthy green or blue-green color at day 14. The inhibition of growth was visible on many algal strains when the CO_2_ level increased to 10 or 20%.

**Figure 3:**
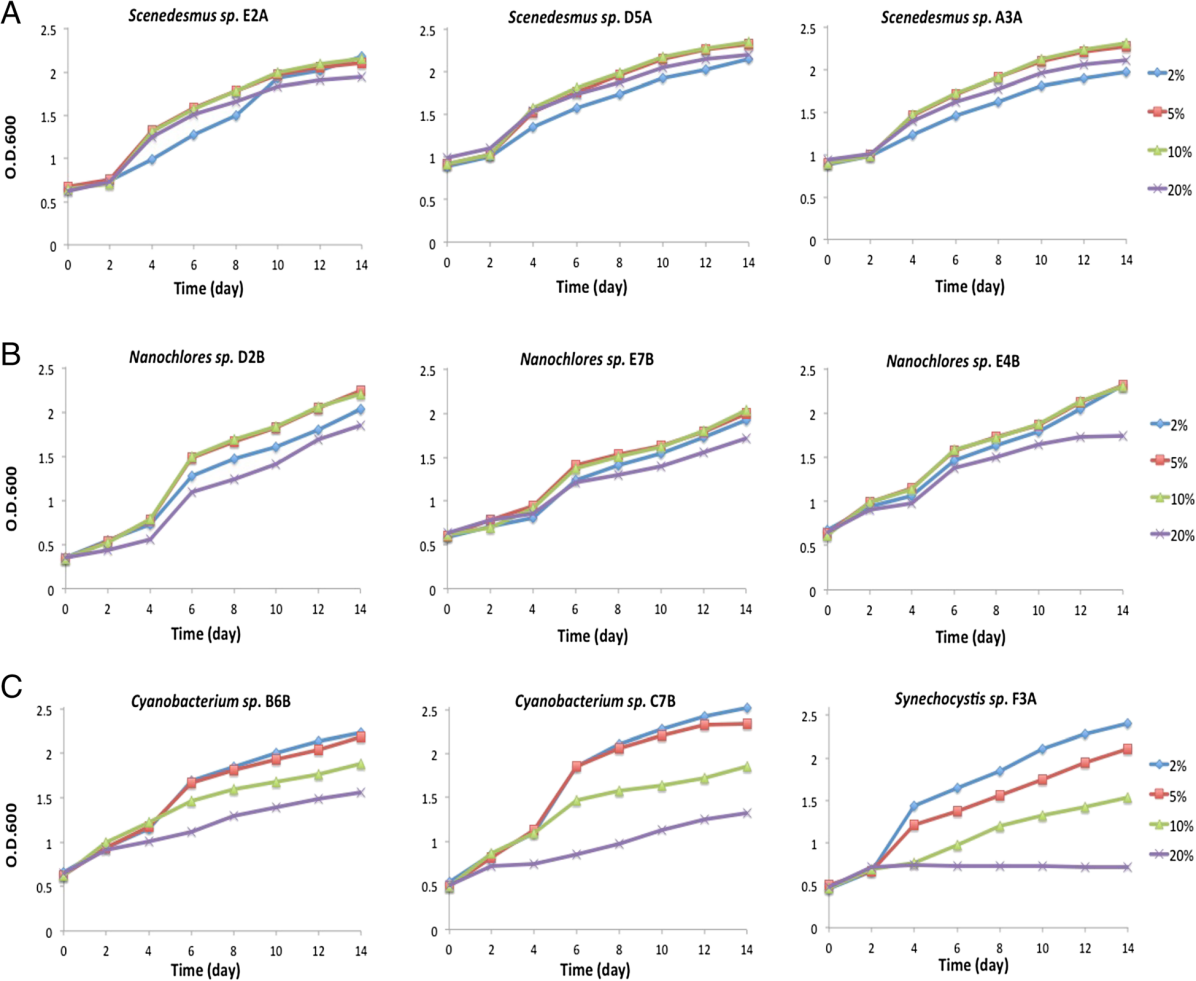
**Time course of OD600 reading for algal strains under 2 (Blue), 5 (Red), 10 (Green) and 20% (Purple) CO**_**2**_**.** Panel **A***Scenedesmus sp.* E2A/D5A/A3A, Panel **B**: *Nannochloris sp*. D2B/E7B/E4B Panel **C**: *Cyanobacterium sp.* B6B, C7B and *Synechocystis sp.* F3A. The strains were named due to their location on microplates.

Growing multiple algal strains in a 48-well or 96-well plate allows a quick measurement and direct comparison of algal cell density using a plate reader. Using the system we developed here, growth performance of all tested strains at different CO_2_ concentrations were monitored. The growth curves allowed us to compare and evaluate the tolerance capacity of selected algae under different CO_2_ conditions. For example, three *Scenedesmus* strains showed a rapid growth under 4 different CO_2_ concentrations (2, 5, 10 and 20%) (Figure 3, panel A). The growths of these algal strains were not affected by increasing CO_2_ level (up to 20%), suggesting that they may tolerate even higher level of CO_2_. In contrast, the growths of three cyanobacterial strains were inhibited when the CO_2_ level was increased to 10 or 20% (Figure 3, panel C). The degree of growth inhibition increased with increasing concentration of CO_2_ suggesting that this system provides sufficient sensitivity for distinguishing algal strains capable of tolerating different levels of CO_2_.

Within all the 96 strains tested, 17 strains were able to maintain similar growth rates with CO_2_ concentration ranging from 2 to 20%, and these algal strains were considered to be high CO_2_ tolerant strains (Figure 2). The algal strains that only grew under 5% CO_2_ concentration were considered as CO_2_ sensitive strains and may not be suitable for the CO_2_ mitigation purpose. In general, the seawater strains tend to show better performance under elevated CO_2_ stress compared with freshwater strains. One explanation would be that seawater medium (SN15) is a better buffering system than freshwater medium (BG11), therefore smaller decrease in pH in the seawater medium poses less acidification stress to microalgae when both media are exposed to the same ambient CO_2_.

**Figure 4:**
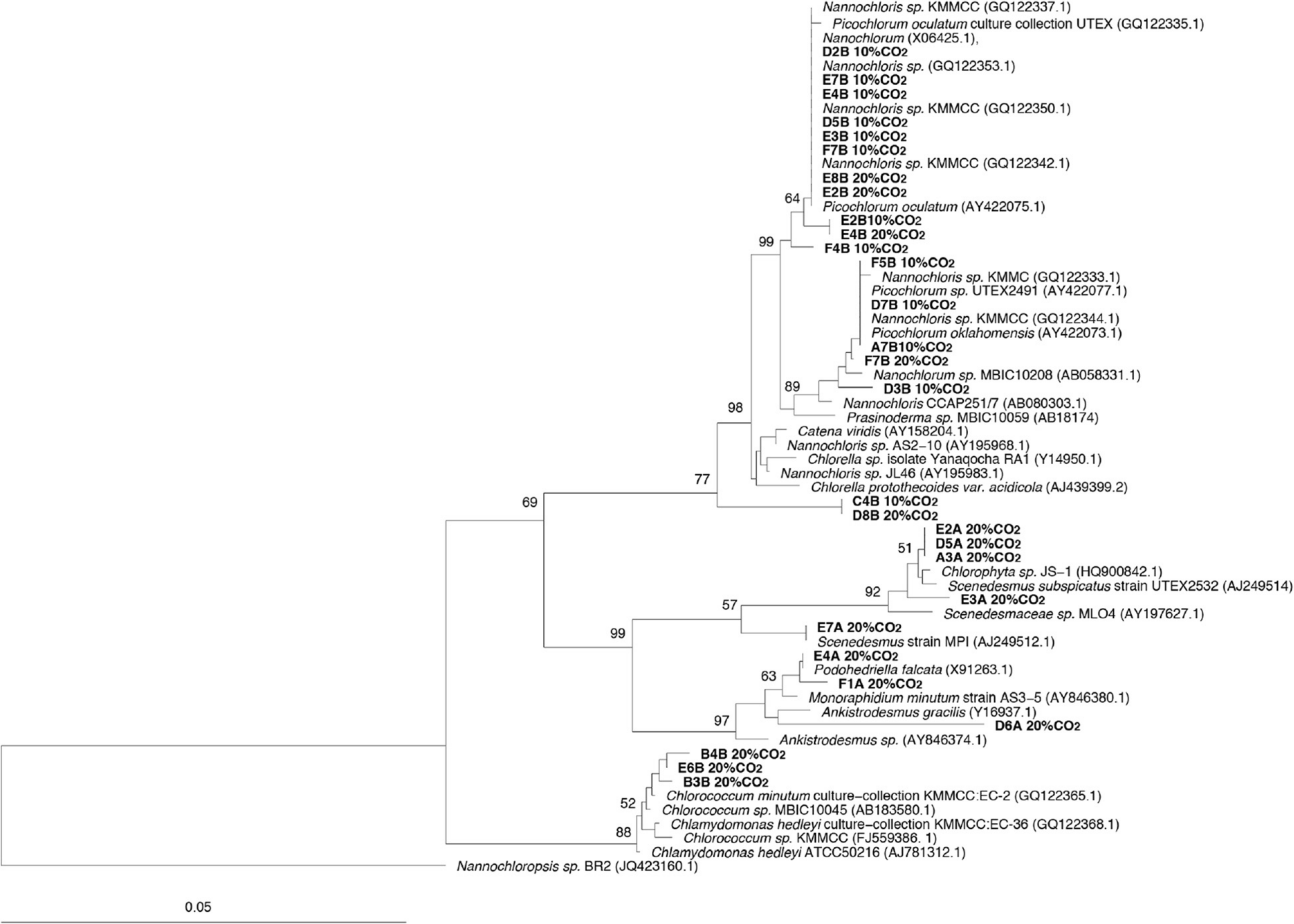
**Phylogenetic tree of CO**_**2 **_**tolerant microalgae based on 18S *rRNA *sequences, *Nannochloropsis *is used as an outgroup.** Bootstrap value = 100.

The algal strains that can tolerate 10 and 20% CO_2_ were identified by sequencing the partial 18S *rRNA* gene or 16S *rRNA* gene. In this study, 5 strains of *Scenedesmus* and 3 strains of *Chlorococcum* can tolerate 20% CO_2_, and 10 strains of *Nannochloris* can tolerate 10% CO_2_. The majority of CO_2_-tolerating strains are closely related to *Scenedesmus sp., Nannochloris sp.* and *Chlorococcum sp.* (Figure 4). Three closely related *Scenedesmus* strains (E2A, D5A, and A3A) showed little effect on their growth when exposed to 2, 5, 10 and 20% CO_2_ (Figure 3, panel A). Growths of three *Nannochloris* strains (D28, E7B, and E4B) were slightly inhibited at 20% CO_2_, but were similar at 2, 5, and 10% CO_2_ (Figure 3, panel B). These results suggest that many microalgae in genera *Scenedemus* and *Nannochloris* can grow under high levels of CO_2_. Other studies have also reported that many algal species from these two genera can tolerate high concentration of CO_2_[5,18,21]. In contrast, tested cyanobacterial strains (B6B, C7B, and F3A) grew poorly under high concentrations of CO_2_ (Figure 3, panel C). Among the limited number of algal strains we tested, it appears that algal species from *Scenedesmus, Nannochloris* and *Chlorococcum* are good potential candidates for sequestering CO_2_ in power plant flue gas.

The majority of the 20% CO_2_ tolerant strains formed several separate branches, represented by genera *Scenedesmus*, *Chlorococum* and *Ankistrodesmus* (Figure 4), suggesting that certain groups or genotypes of algae tend to perform better under high CO_2_ level compare with other algal groups. When more algal strains from diverse taxa were tested for CO_2_ tolerance, the phylogenetic information may provide a useful link to the potential of CO_2_ tolerance of algal strains in the future.

One of *Scenedesmus* strains (E7A) has been tested in large photo-bioreactors (500 L) charged with flue gas (10-12% CO_2_), and it was able to maintain vigorous growth and consume the vast majority of influx CO_2_ (data not published). This test suggests that the algal strains selected using our high throughput system may be suitable for large scale cultivation.

The GasPak system we demonstrated here is designed for high throughput selection of CO_2_ tolerant algal strains. Ideally, it would be useful to integrate a CO_2_ sensor into the system so that the actual concentration of CO_2_ in the GasPak chamber can be monitored. It is possible that the CO_2_ concentration in the chamber could decrease significantly as algae continue to grow over a longer period. Given the fact that algae showed consistent growth trends under different CO_2_ concentrations, we believe that the GasPak system is able to maintain desirable CO_2_ levels during the 2-week experiment.

## Conclusion

We introduced a high throughput system that can be used to quickly select microalgae or other microorganisms that can grow under different concentrations of CO_2_ or other type of gases. Our system provides an adjustable gas input and yields reproducible growth measurements. The growth performance of hundreds of algal strains can be compared at the uniform and sustainable condition using this system. In addition, the system can be used for high throughput screening for algal strains that can tolerate other gases, such as NOx, SOx, or flue gas.

## Methods

### Organisms and culture conditions

The algal strains used in this study were isolated from waters collected from different parts of the Chesapeake Bay including the Baltimore Inner Harbor and the Back River (Baltimore, Maryland), using agar plates made of BG11 [26] as a freshwater medium and SN medium [27] as a seawater medium. Single algal colonies were picked and transferred to 96-well plates, and scaled up to large culture flasks. The algal cultures were illuminated continually using the plant light (Agro-Lite R20, 50 W, PHILIPS) at 25μE/ m^2^/s. This light level was carefully selected for growing algae in the small volume of 48 well Costar plates (Coring, NY, USA). Considering the low starting algal density and the amount of photon received by individual cells, the light intensity in this setting is within the appropriate range.

### The CO_2_ mixing and incubation system

**Figure 5:**
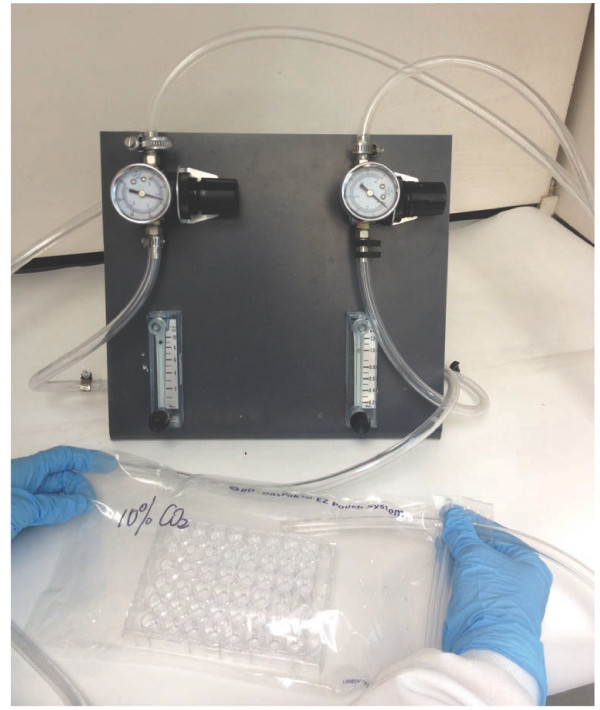
**System used for charging specific concentration of CO**_**2 **_**to the GasPak bags.** For instance, to generate 10% (v/v) CO_2_, the flow of pure CO_2_ was set at the speed of 1 L/min using the controller on the right hand side and the flow of air was set at a velocity of 9 L/min using the controller on the left hand site. The CO_2_/air mixture was then used to inflate the GasPark bag as shown.

To set up different concentrations of CO_2_, pure CO_2_ and air were blended using a device with two gas flow meters (Figure 5).

### CO_2_ equilibrium experiment

In order to test the stability of CO_2_ concentration inside GasPak™ bag (Becton Dickinson, NJ, USA), one milliliter of BG11 or SN15 medium was added to individual wells on a 48-well microtiter plate. Four identical plates were prepared and placed into 4 GasPak™ bags which were charged with 2, 5, 10 and 20% CO_2_, respectively. The pH values in the culture media were measured at 2, 4, 24 and 48 hours, using an Accumet Basic pH meter (Fisher Scientific). Only culture media (no algal inoculation) were used in this test, and the same experiment was repeated 3 times.

### High throughput CO_2_ tolerant strain screening

A 48-well-microtiter plate (without lid) that contains multiple algal strains was placed inside a GasPak™ bag and the bag was aerated with desired concentration of CO_2_. The sealed bags were incubated with light at 25μE/ m^2^/s.

In this experiment, different algal strains were dispensed into 48-well plates and charged with 2, 5, 10 and 20% of CO_2_ respectively. The growth of algae was monitored by cell density (OD600) every other day using a multi-mode microplate reader (Molecular devices, SpectraMax M5). After reading, the culture plates were placed back into the bags, and the system was re-charged with desired concentration of CO_2_.

### Identification of algal strains

Genomic DNA of selected strains were extracted and 18S or 16S ribosomal RNA gene was amplified using the universal primers for eukaryotes and prokaryotes, respectively [28]. Phylogenetic trees were constructed based on partial 18S or 16S *rRNA* gene sequences using ARB Neighbor-joining algorithms with 100 bootstrap [29]. Comparison was carried out between selected strains from this work and high CO_2_ tolerant species reported from other studies.

## Competing interests

The authors have declared that no competing interests exist.

## Authors’ contribution

Experimental design: FC, LZ. Performing the experiments: LZ FZ. Data analysis: FC LZ FZ. Manuscript preparation: LZ FC FZ. All authors read and approved the final manuscript.
